# Costs of maternity leave to support breastfeeding; Brazil, Ghana and Mexico

**DOI:** 10.2471/BLT.19.229898

**Published:** 2020-04-08

**Authors:** Mireya Vilar-Compte, Graciela M Teruel, Diana Flores-Peregrina, Grace J Carroll, Gabriela S Buccini, Rafael Perez-Escamilla

**Affiliations:** a Research Center for Equitable Development EQUIDE, Universidad Iberoamericana, Prolongación Paseo de la Reforma 880, Lomas de Santa Fé, Mexico City, 01219, Mexico.; b Yale School of Public Health, New Haven, United States of America.

## Abstract

**Objective:**

To develop a method to assess the cost of extending the duration of maternity leave for formally-employed women at the national level and apply it in Brazil, Ghana and Mexico.

**Methods:**

We adapted a World Bank costing method into a five-step method to estimate the costs of extending the length of maternity leave mandates. Our method used the unit cost of maternity leave based on working women’s weekly wages; the number of additional weeks of maternity leave to be analysed for a given year; and the weighted population of women of reproductive and legal working age in a given country in that year. We weighted the population by the probability of having a baby that year among women in formal employment, according to individual characteristics. We applied nationally representative cross-sectional data from fertility, employment and population surveys to estimate the costs of maternity leave for mothers employed in the formal sector in Brazil, Ghana and Mexico for periods from 12 weeks up to 26 weeks, the WHO target for exclusive breastfeeding.

**Findings:**

We estimated that 640 742 women in Brazil, 33 869 in Ghana and 288 655 in Mexico would require formal maternity leave annually. The median weekly cost of extending maternity leave for formally working women was purchasing power parity international dollars (PPP$) 195.07 per woman in Brazil, PPP$ 109.68 in Ghana and PPP$ 168.83 in Mexico.

**Conclusion:**

Our costing method could facilitate evidence-based policy decisions across countries to improve maternity protection benefits and support breastfeeding.

## Résumé

**Objectif:**

Développer une méthode permettant de calculer le coût d'une prolongation du congé de maternité pour les femmes officiellement employées au niveau national et l'appliquer au Brésil, au Ghana et au Mexique.

**Méthodes:**

Nous avons adapté une méthode de calcul des coûts empruntée à la Banque mondiale et l'avons divisée en cinq étapes afin d'estimer le coût d'un allongement de la durée du congé de maternité. Notre méthode a utilisé le prix unitaire d'un congé de maternité en s'appuyant sur le revenu hebdomadaire moyen des femmes; le nombre de semaines de congé supplémentaires à analyser pour une année donnée; et la population pondérée de femmes en âge de travailler et de procréer dans un pays donné durant cette année. Nous avons pondéré la population en fonction de la probabilité d'avoir un enfant cette année-là chez les femmes occupant un emploi officiel, selon des caractéristiques individuelles. Nous avons eu recours à des données transversales représentatives à l'échelle nationale issues d'enquêtes sur la fertilité, l'emploi et la population afin de déterminer le coût du congé de maternité des mères travaillant dans le secteur officiel au Brésil, au Ghana et au Mexique. Et ce, sur des périodes comprises entre 12 et 26 semaines, qui correspondent à la durée d'allaitement exclusif recommandée par l'OMS.

**Résultats:**

Nous estimons que chaque année, 640 742 femmes au Brésil, 33 869 femmes au Ghana et 288 655 au Mexique auraient besoin d'un congé de maternité officiel. Le coût hebdomadaire moyen d'un allongement du congé de maternité pour les femmes officiellement employées, exprimé en dollars internationaux à parité de pouvoir d'achat ($PPA), est de 195,07 $PPA par femme au Brésil, 109,68 $PPA au Ghana et 168,83 $PPA au Mexique.

**Conclusion:**

Notre méthode de calcul des coûts pourrait faciliter les décisions politiques fondées sur des données probantes dans les différents pays, afin d'améliorer les avantages liés à la protection de la maternité et de favoriser l'allaitement.

## Resumen

**Objetivo:**

Elaborar un método para evaluar el costo que supone ampliar la duración de la licencia de maternidad de las mujeres empleadas oficialmente a nivel nacional con el fin de aplicarlo en Brasil, Ghana y México.

**Métodos:**

Se adaptó un método de cálculo de costos del Banco Mundial a un método de cinco pasos para estimar los costos relacionados con la ampliación de la duración de los mandatos de licencia de maternidad. El método utilizó el costo unitario de la licencia de maternidad basado en los salarios semanales de las trabajadoras; el número de semanas adicionales de licencia de maternidad que se debían analizar para un año determinado; y la población ponderada de mujeres en edad de procrear y de trabajar legalmente en un país determinado en ese año. Se ponderó la población por la probabilidad de tener un hijo ese año entre las mujeres con empleo formal, según las características individuales. Además, se aplicaron datos transversales representativos a nivel nacional que se obtuvieron de las encuestas de fertilidad, empleo y población para estimar los costos de la licencia de maternidad de las madres empleadas en el sector formal de Brasil, Ghana y México por periodos de 12 a 26 semanas, que es el objetivo de la OMS para la lactancia materna exclusiva.

**Resultados:**

Se estimó que 640 742 mujeres en Brasil, 33 869 en Ghana y 288 655 en México requerirían anualmente una licencia de maternidad formal. El costo semanal medio de la ampliación de la licencia de maternidad para las mujeres que trabajan oficialmente fue de 195,07 dólares internacionales de paridad del poder adquisitivo ($PPA) por mujer en Brasil, 109,68 $PPA en Ghana y 168,83 $PPA en México.

**Conclusión:**

Este método de cálculo de costos podría facilitar la toma de decisiones sobre política basadas en pruebas para mejorar las prestaciones de protección de la maternidad y apoyar la lactancia materna en todos los países.

## ملخص

الغرض وضع طريقة لتقييم تكلفة تمديد مدة إجازة الأمومة للنساء العاملات بشكل رسمي على المستوى الوطني، وتطبيقها في البرازيل وغانا والمكسيك.

الطريقة قمنا بتطويع طريقة حساب التكاليف الخاصة بالبنك الدولي في طريقة من خمس خطوات لتقدير تكاليف تمديد مدة إجازات الأمومة. اعتمدت طريقتنا على تكلفة وحدة إجازة الأمومة على أساس الأجر الأسبوعي للسيدة العاملة؛ وعدد الأسابيع الإضافية لإجازة الأمومة المطلوب تحليلها لسنة ما؛ والعدد المرجح للسيدات في سن العمل الإنجابي والقانوني في بلد ما في تلك السنة. قمنا بتقييم السكان من خلال احتمال إنجاب طفل في تلك السنة بين السيدات في الوظائف الرسمية، وفقًا للخصائص الفردية. قمنا بتطبيق بيانات مستعرضة تمثيلية على المستوى الوطني من مسوح الخصوبة والتوظيف والسكان، وذلك لتقدير تكاليف إجازة الأمومة للأمهات العاملات في القطاع الرسمي في كل من البرازيل وغانا والمكسيك، لفترات من 12 أسبوعاً إلى 26 أسبوعاً، وهو ما يمثل هدف منظمة الصحة العالمية (WHO) للرضاعة الطبيعية الحصرية.

النتائج قمنا بتقدير أن 640742 سيدة في البرازيل، و33869 سيدة في غانا، و288655 سيدة في المكسيك، سوف يطلبون إجازة أمومة رسمية سنوياً. كان متوسط ​​التكلفة الأسبوعية لتمديد إجازة الأمومة للسيدات العاملات بشكل رسمي، بالدولار الدولي وفقاً لتعادل القوى الشرائية ($PPP)، هو 195.07 $PPP لكل امرأة في البرازيل، و109.68 $PPP في غانا، و168.83 $PPP في المكسيك.

الاستنتاج يمكن أن تسهل طريقة التكلفة لدينا من اتخاذ قرارات للسياسات المستندة على الأدلة عبر الدول، لتحسين

## 摘要

**目的:**

旨在从国家层面制定一种方法来评估延长有正式工作的女性的产假期限所产生的成本，并将该方法在巴西、加纳和墨西哥实施。

**方法:**

我们将世界银行的成本计算方法调整为五步法，用以估算延长产假期限的成本。我们的方法采用根据就业女性的每周工资计算得到的单位产假成本；在某年中需分析的额外产假周数；以及某国当年处于育龄和法定工作年龄妇女的加权人口。根据个体特征，我们依据有正式工作的女性当年生育婴儿的机率对人口进行了加权。我们从生育、就业和人口调查中选取具有全国代表性的横断面数据，估计巴西、加纳和墨西哥有正式工作的母亲产假期限从 12 周到 26 周（世卫组织纯母乳喂养的目标）所产生的成本。

**结果:**

我们估计巴西、加纳和墨西哥每年需要正式产假的女性分别为 640,742 人、33,869 人和 288,655 人。巴西、加纳和墨西哥延长每位有正式工作女性的产假所产生的每周成本中位数分别为购买力平价国际美元 (PPP$) 195.07、PPP$ 109.68 和 PPP$ 168.83。

**结论:**

我们的成本计算方法可以促进各国落实以证据为基础的政策决定，改善生育保障福利并支持母乳喂养。

## Резюме

**Цель:**

Разработка метода для оценки затрат на национальном уровне на увеличение продолжительности отпуска по беременности и родам для официально работающих женщин и его применение в Бразилии, Гане и Мексике.

**Методы:**

Авторы адаптировали метод оценки Всемирного банка и превратили его в пятиступенчатую методику для оценки затрат, связанных с увеличением продолжительности отпуска по беременности и родам. В данной методике использовалась удельная стоимость отпуска по беременности и родам, основанная на сумме еженедельной заработной платы работающих женщин, количество дополнительных недель отпуска по беременности и родам анализировалось для конкретного года, а также рассчитывался взвешенный показатель женского населения репродуктивного возраста в группе разрешенного законом возраста для трудоустройства для данного года. Авторы взвесили численность населения по вероятности рождения ребенка в данном году среди официально трудоустроенных женщин в соответствии с индивидуальными характеристиками. Для оценки стоимости отпуска по беременности и родам для официально трудоустроенных матерей в Бразилии, Гане и Мексике на период от 12 до 26 недель (целевой показатель ВОЗ для исключительно грудного вскармливания) применялись репрезентативные в национальном масштабе перекрестные данные, полученные в ходе опросов, связанных с фертильностью, занятостью и численностью населения.

**Результаты:**

По оценкам авторов, примерно 640 742 женщины в Бразилии, 33 869 женщин в Гане и 288 655 женщин в Мексике ежегодно нуждаются в официальном отпуске по беременности и родам. Средняя недельная стоимость продления отпуска по беременности и родам для официально трудоустроенных женщин в пересчете на международный доллар паритетной покупательной способности (ППС$) составила 195,07 на одну женщину в Бразилии, 109,68 в Гане и 168,83 в Мексике.

**Вывод:**

Предложенный метод расчета затрат может способствовать принятию обоснованных политических решений в разных странах в целях повышения эффективности охраны материнства и поддержки грудного вскармливания.

## Introduction

Creating an enabling environment for women to successfully breastfeed has wide-reaching health, economic and environmental benefits.^1^^,^^2^ Improving breastfeeding outcomes globally could prevent an estimated 823 000 child deaths and 20 000 breast cancer deaths every year.^1^ However, the prevalence of exclusive breastfeeding among infants younger than 6 months remains low, around 37% globally.^3^

Breastfeeding practices are affected by a wide range of factors, including sociocultural and economic contexts, health systems, families and communities, employment, and individual attributes of the mother, the infant and their relationship.^2^ Interventions in these areas can potentially promote a more enabling environment, and in turn, achieve the global World Health Organization (WHO) target of 70% of babies exclusively breastfed up to 6 months by 2034.^4^^,^^5^ Public policies are needed, especially for women such as working mothers who may be deterred from breastfeeding. Given the increase in women’s participation in the labour market around the world, maternity protection policies are considered essential for improving breastfeeding practices.^6^

Giving women a period of paid absence from work after childbirth provides social, developmental and health benefits for working mothers and their children and has been shown to be effective for increasing exclusive breastfeeding.^2^^,^^7^^,^^8^ Evidence from Brazil, Canada, China, Sweden and the United States of America suggests that the duration of maternity leave has a positive association with exclusive breastfeeding and maintenance of breastfeeding.^6^^,^^9^^–^^14^ A study that assessed the expansion of the maternity and parental leave mandate in Canada from 25 to 50 weeks found a significant increase in exclusive breastfeeding rates at 6 months by 5.8 percentage points.^6^^,^^14^ Evidence from Sweden reveals that long periods of mandated maternity leave promote higher rates of breastfeeding and a larger share of women returning to work: both important factors for social well-being and development.^6^ Recent evidence from 38 low- and middle-income countries showed that the extension of maternity leave has the potential to reduce barriers to breastfeeding for working mothers.^8^ In addition, the length of maternity leave is associated with improved mother’s mental health,^15^^,^^16^ and lower neonatal and postnatal mortality.^16^

Previous studies have highlighted work-related issues as a major reason why mothers do not start breastfeeding or stop exclusive breastfeeding early.^10^ The effects of work on women’s decisions to breastfeed are multidimensional, including fatigue and financial stress.^2^^,^^6^ Hence, labour protection policies have a strong potential to positively influence both breastfeeding and women’s labour market participation.^13^ Although many countries have maternity protection legislation, only 99 (out of 185) meet or exceed the minimal 14 weeks of paid maternity leave recommended by the International Labour Organization (ILO),^17^ 57 countries meet 14–17 weeks of leave, and just 42 countries meet or exceed 18 weeks leave. These numbers imply that employed women globally face inadequate maternity protection to enable them to achieve their breastfeeding goals.^2^

Maternity leave can be financed in different ways: social security schemes that rely on a mix of contributions from employers, employees and government funds; public funds; or solely by the employer. To effectively scale up and sustain coverage of effective breastfeeding interventions, the costs must be considered,^2^ specifically at the country level.^18^ Identifying the economic implications of breastfeeding should be a priority, as increasing breastfeeding prevalence could have substantial economic effects,^19^ for example, on a country’s gross domestic product. Previous studies have highlighted the need for standardized breastfeeding costing frameworks at the national level.^18^^,^^20^^,^^21^ Global costing frameworks for breastfeeding have helped highlight the need for further investment and resources.^22^^,^^23^ However, these methods have seldom been adopted at the national level to estimate the costs of maternity leave policies that could be used by local breastfeeding advocates and policy-makers.

Previous studies have estimated the costs of extending the duration of maternity leave for women employed in the formal sector in Chile,^24^ Indonesia^25^ and Norway^26^ and the cost of implementing new maternity schemes in the USA.^20^ Despite the relevance of these specific costing studies, there is a need for pragmatic, standardized algorithms for establishing the costs of incrementally expanding the duration of mandates at the country level. Governments can then assess the financial feasibility of implementing or expanding programmes. Given that the cost of extending maternity leave can vary greatly across countries due to differences in policies and wages, it is important to develop a method that uses data commonly available across countries. The aim of our study was to develop a method for estimating the cost of extending the duration of maternity leave for mothers employed in the formal sector at the national level using existing country-specific data and apply it in Brazil, Ghana and Mexico.

## Methods

### Setting

We used nationally representative, publicly available, cross-sectional data from each country. While the data were comparable across countries, the dates of data collection were different; data for Brazil were collected in 2015, Ghana in 2017 and Mexico in 2013–2014. These countries were selected because they are diverse across several domains: economic development, labour market structure, women’s participation in the labour force, fertility rate and breastfeeding indicators (Table 1). Furthermore, regulations on maternity leave differ. In Brazil, female employees receive mandatory maternity leave at full pay for about 4 months, paid by the social security agency, while employers have the option of offering an additional 2 months and deducting the amount paid from its corporate income tax.^29^ In Ghana, female workers are entitled to a full period of paid maternity leave of at least 12 weeks, which is paid by the employer.^31^ Mexico has extended the maternity leave mandate at full pay from 12 to 14 weeks, financed by the social security system.^29^

**Table 1:** Background socioeconomic characteristics of the studied countries

Variable	Brazil	Ghana	Mexico
Total population, no.	207 833 831	29 121 471	124 777 324
GDP per capita, PPP$	14 236	4 051	17 956
Informal employment, % of total employment in 2015^a^	38.3	83.2	60.7
Working-age population, no.^b^	144 882 359	17 219 574	82 377 995
No. (%) of working-age women	73 366 432 (69.5)	8 495 756 (59.1)	42 478 203 (66.6)
Population of women, no. (%)	105 601 740 (50.8)	14 366 668 (49.3)	63 752 822 (51.1)
Fertility rates, total births per woman	1.7	3.9	2.2
Current duration of maternity leave^c^	120 days (about 17 weeks)	12 weeks	14 weeks
Exclusive breastfeeding, % of children aged under 6 months in 2014^d^	39.0	52.1	30.1

GDP: gross domestic product; PPP$: purchasing power parity constant 2011 international dollars.

^a^ Informal employment is based on a harmonized measure of the International Labour Organization (ILO). The information for Brazil and Ghana is reported in the World Development Indicators,^27^ and we obtained the data for Mexico from the ILO.^28^

^b^ Working age was defined as 15–64 years old.

^c^ Data were from the ILO 2014.^29^ The Mexico Federal Labour Law was modified to 14 weeks in September 2019; before this maternity leave was for 12 weeks.

^d^ Data for Ghana and Brazil were obtained from the World Development Indicators^27^ and for Brazil from the Global Breastfeeding Collective.^30^

Data sources: World Development Indicators 2017^27^ (unless otherwise specified).

### Costing method 

We adapted a costing method from the World Bank,^18^^,^^23^ which estimates the financial needs for scaling up a nutrition intervention to achieve World Health Assembly global nutrition targets.^32^ The method is based on the following equation:


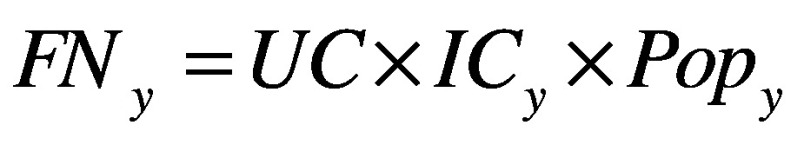
(1)

where *FN_y_* is the annual financial need for a given intervention in year *y*, *UC* the unit cost, *IC_y_* is the incremental coverage (*IC*), assumed for year *y* and *Pop_y_* is the target population in year *y.*

We modified this costing approach to make it more precise and suitable to maternity leave mandates. We weighted the population by *α*, which is the probability of having given birth among formally employed women according to the following characteristics: age, marital status, educational level and locality (urban or rural). Hence, we estimated the cost of extending the maternity leave for women working in the formal sector as:


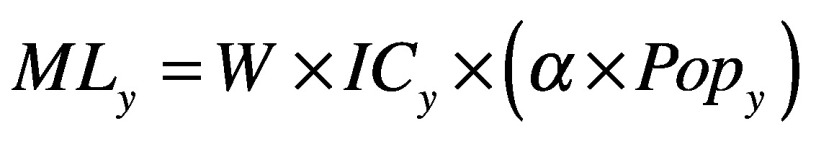
(2)

Where *ML_y_* is the maternity leave cost needed for a given year of intervention, *W *is the maternity leave unit cost, *IC_y_* is the weekly incremental coverage for maternity leave assumed for year *y* and *α × Pop_y_* is the population of women of reproductive and legal working ages in a given country in year *y* weighted by *α* (probability of having given birth according to women’s characteristics).

A key aspect behind this modelling approach is that it is based on five clearly delineated steps that could be replicated across countries (Table 2). To apply this method, nationally representative surveys with data on employment and fertility should be available, and demographic data are required to adequately calibrate to the population size. These are data sources commonly available in different countries.

**Table 2:** Steps for estimating the annual costs of extending maternity leave for women in formal employment in Brazil, Ghana and Mexico

Step	Aim	Data used	Process	Variables input	Notes
Step 1	Compute the probability of women having a baby in the previous year, given a set of women’s characteristics, needed to compute the value of *α* in Equation 2 in the methods section	Fertility data Brazil: National Household Sample Survey 2015^33^ Ghana: Ghana Living Standard Survey 2017^34^ Mexico: National Survey of Demographic Dynamics 2014^35^	Identify women of reproductive age. Among this subset of women, generate combinations according the available sociodemographic variables. For each of the combinations, calculate the percentage of women who had a baby in the previous year (as a proportion of the total number of women of reproductive age)	Reproductive age Brazil & Ghana: 16–49 years; Mexico: 18–49 years. Marital status Brazil & Ghana: single; married or living with partner; widow or divorced or separated; Mexico: single; married; divorced. Educational level Brazil: no education; kindergarten or incomplete primary; complete primary or incomplete middle; complete middle or incomplete high school; complete high school; higher or any technical career. Ghana: no education; primary or kindergarten; secondary or middle or incomplete high school; complete high school or higher incomplete or technical career; higher complete or more. Mexico: incomplete primary or less; primary or some secondary; secondary or some high school; high school completed; technical training or incomplete professional education; university degree. Locality Brazil & Ghana: rural; urban. Mexico: rural; semi-urban; urban.	Number of combinations Brazil: 180 Ghana: 150 Mexico: 270
Step 2	Estimate the probability of women working in the formal sector having a baby in the previous year (variable *α*), given a set of women’s characteristics	Fertility and employment data Brazil: National Household Sample Survey, 2015^33^ Ghana: Ghana Living Standard Survey, 2017^34^ Mexico: National Survey of Demographic Dynamics, 2014^35^ and the National Survey of Occupation and Employment, 2013–2014^36^	Define formal employment. Considering the combinations generated in Step 1, add employment information to estimate the probability of having a baby only among formally employed women. This may be done by tabulating data from a single survey (such as in Brazil and Ghana) or through merging different data sets (as in Mexico)	Formal employment Brazil: women with a formal contract, including domestic workers, military and civil servants, as well as employers and self-employed persons who contribute to social security (variables to operationalize: occupation and social security contribution). Ghana: women who have at least one social benefit (maternity leave, sick leave or holidays) and a written or verbal contract (variables to operationalize: holidays, paid leave and contract). Mexico: women who have access to social security and have the right to a paid maternity leave (variable to operationalize: social security)	NA
Step 3	Estimate the population of women of reproductive age, weighted by the probability of having a baby in the previous year based on individual characteristics (*α* * × Pop_y_*). This step seeks to generate a more realistic estimate of the women employed in the formal sector who may claim maternity leave in a given year	Census data or demographic projections. Brazil: World Bank 2015 population projections for age group^37^ Ghana: World Bank 2017 population projections for age group^37^ Mexico: Inter-census Mexican Survey, 2015^38^	Identify national estimates of women in reproductive ages *Pop_y_* Multiply the population by each of the values of *α’s* generated in Step 2	No additional variables	While some surveys used in Steps 1 and 2 may have expansion factors (e.g. Brazil), we strongly recommend not using them as they were generated for expanding other population subgroups. This may increase the error of any estimated parameter
Step 4	Estimate the mean or median weekly wages of women working in the formal sector, given a set of women’s characteristics (*W*). Multiply the wage by the weighted population of women of reproductive age	Employment or wage data. Brazil: National Household Sample Survey 2015^33^ Ghana: Ghana Labour Force Survey 2015^39^ Mexico: National Survey of Occupation and Employment 2013–2014^36^	For each group of women (combinations) identify the mean or median weekly wage. To decide whether to use the mean or the median, plot a density function graph of weekly wages to see if its distribution is symmetrical (see Fig. 1 for example). If the distribution is not symmetrical and the mean is not centred, use the median. Determine the percentage of the salary that would be covered by the maternity leave benefit and multiply it by the weekly wage. Multiply the covered wage by the weighted population computed in Step 3. To estimate the mean and median weekly cost per woman, *W* × (*α* * × Pop_y_*) can be divided by the estimated number of women expected to receive maternity leave	Weekly wages Brazil: full-time weekly wages (at least 44 hours of work per week). Ghana: full-time weekly wages (at least 40 hours of work per week). Mexico: full-time weekly wages (at least 40 hours of work per week)	The assumption for the three countries was that maternity leave benefits would cover 100% of the salaries
Step 5	Determine the incremental weekly coverage of the maternity leave *IC* according to relevant thresholds. Estimate the annual cost of expanding maternity leave	Laws, international and national organization documents establishing length of maternity leave coverage	Multiply the number of weeks to be covered by *W* × (*α* * × Pop_y_*) to estimate the annual cost of the expansion in the maternity leave coverage	NA	NA

NA: not applicable.

### Application of costing method

Following the steps of the costing method (Table 2), we estimated the annual costs of extending maternity leave for formally employed women in Brazil, Ghana and Mexico.

Step 1 was determining the number of women of reproductive and legal working age who reported having a child in the previous year; this number is necessary for computing *α*. Table 2 summarizes the data sources on fertility for each country. We categorized women of reproductive age according to their age bracket, marital status, educational level and urban or rural residential locality. While the goal was to have a process as standardized as possible, the definitions of the variables slightly differed across countries due to differences in definitions attributable to each country. This led to a different number of possible combinations of women’s characteristics, which derived from the demographic features of each country. For each combination, we assessed the proportion of women who reported having given birth in the previous year. For example, in Brazil the proportion of women aged 30–34 years, who had completed high school, lived in an urban locality and were married, and who had a baby in the previous year, was 8.1%.

Step 2 was to determine the probability of a woman working in the formal sector having had a baby in the previous year (*α*). This step required defining formal employment (Table 2 presents country definitions). Then, using the combinations generated in Step 1, employment information was applied to estimate the probability of having had a baby only among formally employed women. This step required linking fertility and employment data for each of the combinations estimated in Step 1. Hence, the probability of having a baby and working in the formal sector was estimated for each of the combinations.

Step 3 was to identify the target population *Pop_y_* (women of reproductive and legal working ages) through national population estimates (census data and population projections). The national population of women of reproductive age was then weighted (multiplied) by each of the values of *α* estimated in Step 2, expressed as *α* *× Pop_y_*.

**Fig. 1:**
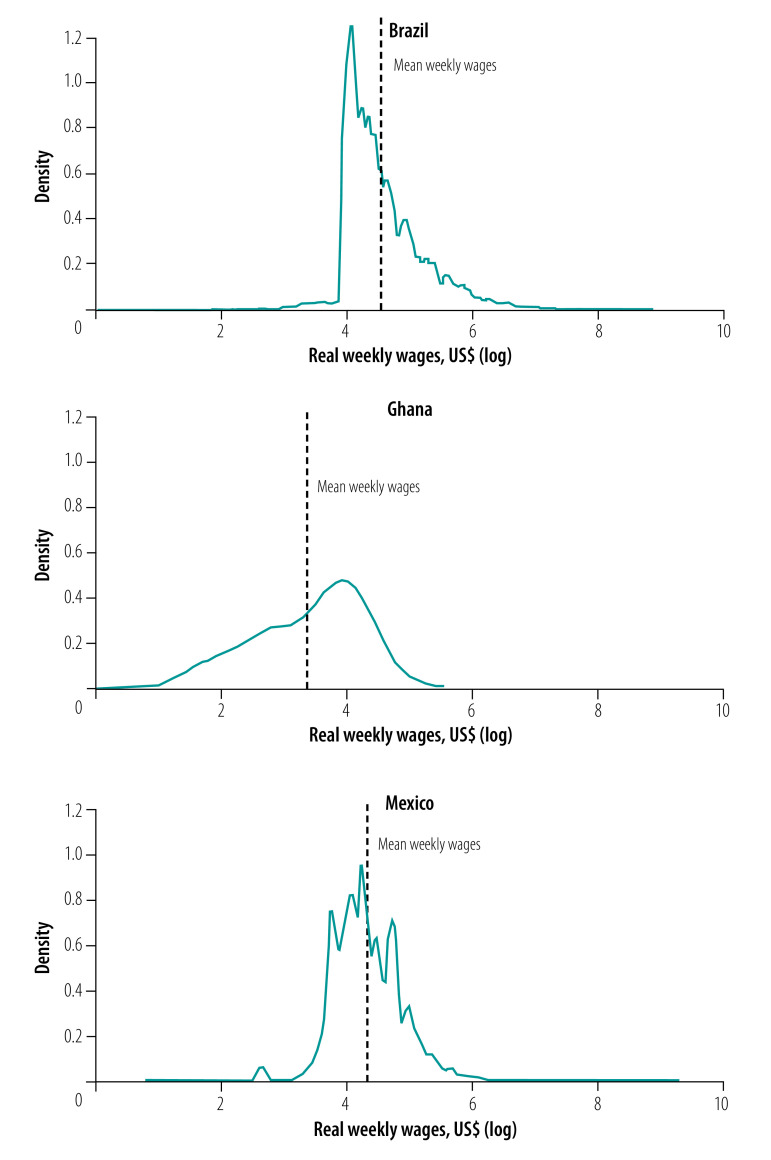
Density function graphs for real weekly wages in Brazil, Ghana and Mexico

Step 4 was to identify the weekly wages of women working in the formal sector (*W*). We estimated *W* for each of the women’s subgroups (based on combinations of their personal characteristics) and operationalized through the weekly wage in United States dollars (US$). The value of *W* was then multiplied by the weighted population *W* × (*α* *× Pop_y_*). More specifically, outcomes of the weighted population obtained through Step 3 (*α* *× Pop_y_*) were multiplied by their corresponding mean or median formal sector wage. Given that wages tend to have skewed distributions (Fig. 1), we estimated mean and median wages. For example, the mean wage of women aged 30–34 years in Mexico with no education, living in a rural locality, married and who had a baby in the previous year was US$ 48.5 per week. An important assumption in this step is that maternity leave covers 100% of the salary, but this can be tailored to country’s specific context (Table 2). The weekly mean and median costs per woman were calculated by dividing cost per week by the estimated number of women expected to receive the maternity leave.

US$: United States dollars in 2018.

Notes: We used data from the National Household Sample Survey 2015 for Brazil;^33^ Ghana Labour Force Survey 2015;^39^and the Mexican National Survey of Occupation and Employment 2013–2014.^36^ The dotted line shows mean weekly wages.

In Step 5 we determined the number of weeks of maternity leave to be assessed (*IC*). We assessed four relevant cut-off points: (i) 12 weeks, which is the number of weeks covered by the formal sector maternity leave in Ghana and Mexico (up to September 2019);^40^ (ii) 14 weeks, which is the minimum duration recommended by the ILO;^41^ (iii) 18 weeks, which is the length of maternity leave coverage currently being discussed by key stakeholders in Ghana; and (iv) 26 weeks, which is consistent with the WHO recommendation of exclusive breastfeeding for the first 6 months of life.^4^ We present estimates for these proposed durations, but the method can be applied for any number of weeks.

All costing calculations were estimated in US$ and PPP$ using 2018 as the reference year using Stata, version 15 (StataCorp, College Station, USA).

### Assessing validity and affordability

To assess the validity of our estimates, we compared our values with those obtained from the administrative records of the Mexican Institute of Social Security. These records represent the real costs incurred for the current maternity leave of working mothers in the formal sector. We restricted the Mexican sample to women affiliated with the social security system, which covers 77.8% (111 838 of 143 797) of formally employed women. We then applied the costing method using the selected population and compared the mean costs obtained with those reported from the Institute’s public registries, corresponding to a maternity leave of 12 weeks in 2014.^42^

In addition, to assess the feasibility of extending maternity leave for women working in the formal sector, we accessed supplementary data for Mexico. We compared the estimated mean cost of one additional week per woman with the weekly cost per child of the social security system’s day-care services and with the weekly cost of feeding an infant with formula milk, if the woman is not breastfeeding.

## Results

The unweighted survey estimates of the total numbers of women in formal employment in Brazil, Ghana and Mexico were 31 665 725 and 143 798, respectively in the relevant year. Table 3 presents the characteristics of these women and the estimated numbers and proportions who gave birth in the previous year. Table 4 summarizes the population of women who would receive maternity leave benefits. According to estimates from our model, the numbers vary due to differences between countries in the population, share of women in the labour force and proportion of women in formal employment. For example, we estimated that 640 742 women in Brazil, 33 869 in Ghana and 288 655 in Mexico would have been granted maternity leave annually. 

**Table 3:** Characteristics of women of reproductive age in formal employment in Brazil, Ghana and Mexico

Variables by country	Total no. of women	Women in formal employment	
		Estimated total no.	Estimated no. (%) giving birth in previous year
**Brazil**			
Age, years			
16–24	8 704	5 112	322 (6.3)
25–29	7 710	5 148	299 (5.8)
30–34	8 948	5 932	261 (4.4)
35–39	8 929	5 742	132 (2.3)
40–49	15 224	9 731	39 (0.4)
Education level			
No education	1 272	533	11 (2.1)
Kindergarten or incomplete primary school	2 853	1 051	39 (3.7)
Complete primary or incomplete middle school	4 247	1 857	87 (4.7)
Complete middle or incomplete high school	7 374	3 723	156 (4.2)
Complete high school	20 336	13 973	377 (2.7)
Higher education or any technical career	13 433	10 528	484 (4.6)
Marital status			
Single	17 121	10 797	259 (2.4)
Married or living with partner	28 113	18 004	936 (5.2)
Widowed or divorced or separated	4 281	2 864	95 (3.3)
Locality			
Urban	45 697	30 064	1142 (3.8)
Rural	3 818	1 601	56 (3.5)
**Ghana**			
Age, years			
16–24	2 481	113	4 (3.5)
25–29	1 631	200	14 (7.0)
30–34	1 683	184	10 (5.3)
35–39	1 524	113	9 (8.0)
40–49	2 533	115	2 (1.5)
Education level			
No education	2 963	18	0 (0.0)
Primary or kindergarten school	1 840	21	2 (8.9)
Secondary or middle or incomplete high school	3 478	101	4 (3.5)
Complete high school or higher education incomplete or technical career	1 422	457	34 (7.5)
Higher education complete or more	149	128	4 (2.8)
Marital status			
Single	2 429	277	5 (1.8)
Married or living with partner	6 379	388	38 (9.9)
Widowed or divorced or separated	1 044	60	0 (0.0)
Locality			
Urban	3 675	511	34 (6.6)
Rural	6 177	214	6 (3.0)
**Mexico**			
Age, years			
18–24	59 065	25 570	1 457 (5.7)
25–29	51 177	27 082	1 598 (5.9)
30–34	50 850	25 821	1 394 (5.4)
35–39	51 781	24 709	914 (3.7)
40–49	88 462	40 615	2 030 (0.5)
Education level			
Incomplete primary school or less	4 495	381	11 (2.9)
Primary or some secondary school	43 113	9 436	274 (2.9)
Secondary or some high school	97 290	36 635	1 465 (4.0)
High school complete	51 465	26 492	1 086 (4.1)
Technical or incomplete professional training	35 810	19 997	620 (3.1)
University degree	69 162	50 855	2 136 (4.2)
Marital status			
Singe	108 169	56 005	840 (1.5)
Married	163 097	73 012	4 308 (5.9)
Divorced	30 069	14 779	443 (3.0)
Locality			
Urban	198 357	107 711	4 093 (3.8)
Semi-urban	40 260	16 962	695 (4.1)
Rural	62 718	19 124	860 (4.5)

Notes: We based Brazil estimates on data from the National Household Sample Survey 2015.^33^ Ghana estimates were based on Ghana Living Standard Survey 2017.^34^ Mexico estimates were based on the National Survey of Occupation and Employment 2013–2014^36^ and National Survey of Demographic Dynamics 2014.^3^

**Table 4:** Estimated costs of annual maternity leave for women in formal employment in Brazil, Ghana and Mexico

Variable	Brazil	Ghana	Mexico
**Population of eligible women^a^**	640 742	33 869	288 655
**Marginal cost per week**			
In PPP$			
Mean	159 342 770	3 747 395	56 245 792
Median	124 989 350	3 714 614	48 734 530
In US$			
Mean	82 078 320	1 714 494	27 756 010
Median	64 382 688	1 699 496	24 049 374
**Total annual cost per 12 weeks leave**			
In PPP$			
Mean	1 912 113 240	44 968 740	674 949 504
Median	1 499 872 200	44 575 368	584 814 360
In US$			
Mean	984 939 840	20 573 929	333 072 120
Median	772 592 256	20 393 956	288 592 488
**Total annual cost per 14 weeks leave**			
In PPP$			
Mean	2 230 798 780	52 463 530	787 441 088
Median	1 749 850 900	52 004 596	682 283 420
In US$			
Mean	1 149 096 480	24 002 917	388 584 140
Median	901 357 632	23 792 948	336 691 236
**Total annual cost per 18 weeks leave**			
In PPP$			
Mean	2 868 169 860	67 453 110	1 012 424 256
Median	2 249 808 300	66 863 052	877 221 540
In US$			
Mean	1 477 409 760	30 860 894	499 608 180
Median	1 158 888 384	30 590 933	432 888 732
**Total annual cost per 26 weeks leave**			
In PPP$			
Mean	4 142 912 020	97 432 270	1 462 390 592
Median	3 249 723 100	96 579 964	1 267 097 780
In US$			
Mean	2 134 036 320	44 576 847	721 656 260
Median	1 673 949 888	44 186 904	625 283 724
**Cost per week per woman**			
In PPP$			
Mean	248.68	110.64	194.85
Median	195.07	109.68	168.83
In US$			
Mean	128.10	50.62	96.16
Median	100.48	50.18	83.32

PPP$: purchasing power parity international dollars; US$: United States dollars in 2018.

^a^ Estimated number of women who would receive maternity leave.

Notes: We based Brazil estimates on data from the National Household Sample Survey 2015,^33^ the Brazil 2010 Census^43^ and World Bank population projections for women age 16–49 years in Brazil from 2010–2015. Ghana estimates were based on Ghana Living Standard Survey 2017,^34^ Ghana Labour Force Survey 2015,^39^ Ghana 2010 Census^44^ and World Bank population projections for women aged 16–49 years from 2010–2017.^37^ Mexico estimates were based on the National Survey of Occupation and Employment 2013–2014^36^ and National Survey of Demographic Dynamics 2014.^35^

Table 4 also summarizes the total cost of maternity leave, considering different lengths of maternity leave (12, 14, 18 and 26 weeks). The costs are presented as both means and medians. Adding an extra week of maternity leave in Brazil would lead to an annual median cost of purchasing power parity international dollars (PPP$) 195.07 per woman. In Ghana the estimated costs were lower (PPP$ 109.68 per woman), while in Mexico costs were closer to those estimated in Brazil (PPP$ 168.83).

The validity analysis we performed with data from Mexico suggested that our costing method under-reported actual costs by about 10% (Table 5). The mean weekly cost of maternity leave per woman in the social security system estimated by our costing method was US$ 96.15 compared with reported costs of US$ 104.73. Our estimated amount is close to the amount resulting from adding the weekly cost per child of the social security day-care services (US$ 56)^45^ plus the weekly cost of provision of infant formula milk (US$ 39).^46^

**Table 5:** Comparison of estimated and reported costs of maternity leave for formally employed women affiliated with the social security system in Mexico

Variable	Estimated	Reported^b^
Population of eligible women, no.^a^	224 487	230 264
Total annual cost of 12 weeks leave, US$	259 030 188	289 409 798
Cost per week per woman, US$	96.15	104.73

US$: United States dollars in 2018.

^a^ Number of women who receive maternity leave.

^b^ Reported by the Mexican Institute for Social Security.

Notes: We based estimates on data from the National Survey of Occupation and Employment 2013–14,^36^ National Survey of Demographic Dynamics 2014,^35^ Mexican Institute for Social Security data^42^ and Intercensus Population Survey.^38^

## Discussion

This study fills a research gap by developing a replicable method to estimate the annual costs of extending maternity leave for women employed in the formal economy. Our approach built upon and extended the application of an accepted and widely used World Bank costing method.^23^ The analysis suggests that estimates from the five-step method were feasible in three different countries from two different regions (Latin America and sub-Saharan Africa) and different income levels (lower-middle and upper-middle income). The replicability of the method is important, as it suggests that costing a maternity benefit for women employed in the formal economy is feasible using data commonly available across countries through existing national sociodemographic and employment surveys, as well as census data. In each country the data sources were different, but the variables for estimation were comparable. It is important to highlight that the accuracy of the costing method will depend on the quality of the survey data of each country and so it is relevant to perform calculations of data quality before embarking on cost estimates. If the data are of adequate quality, we expect that our costing method will facilitate evidence-informed policy decisions across countries to improve maternity protection benefits and potentially improve breastfeeding and other maternal, child and family health outcomes.

Our method was validated by comparing our estimates with actual expenditures observed in Mexico. Similar validations could not be performed for the other two countries due to limitations of the available data. Investigators applying our method in other countries should make comparisons with observed expenditures, as we did in Mexico, to further validate the method in additional settings.

The current research has some limitations. First, despite our efforts to standardize the costing method, there were differences in the national-level surveys, such as different time periods of data collection and the way surveys were structured. We therefore used slightly different data sources in each country. However, nationally representative data were available to estimate the relevant parameters. Another limitation in the standardization was that the difference between countries in definitions of some variables (such as education) led to different categorizations across countries. While the specific categories for each group are not strictly comparable across the three countries, the method leads to estimates that are applicable and valid to each particular context. 

Due to the scope of the costing method, we aimed to estimate aggregate national level costs. Every country will need to do further adaptations in using the costing method to the institutional nature of national maternity leave schemes (such as contributory or tax-funded) and this calls for future research in this area. Similarly, although our analyses did not compare women employed in the public and private sector, our method can easily be extended to conduct such comparative analyses. This analysis would require cutting part of the data to the sub-population of interest; hence it is important to understand how dropping part of the data would affect the statistical power of the sub-analyses. Our analysis estimates the cost of extending maternity leave at a country level based on observed salaries and based on the assumption that the opportunity cost of women is similar between sectors. 

Finally, the analysis was based on countries from the Latin American and sub-Saharan Africa regions and needs to be tested in additional areas including Asia, Europe and North America. While the current analyses focused on costing the extension of maternity leave mandates for women employed in the formal sector, in many low- and middle-income countries women are more likely to work in the informal economy. It is important to also develop costing methods to provide maternity benefits to these women.^47^

While maternity leave protection is a key policy to promote and support breastfeeding for working women, there are other fundamental areas that should also be addressed, such as workplace policies, child care and paternal involvement. Protecting and supporting breastfeeding working mothers requires an integral strategy of which maternity leave mandates are a fundamental part. Supportive labour market policies, such as maternity leave, are essential in high-, middle- and low-income countries if increased breastfeeding rates are to be achieved alongside the participation of women in the labour force.

Further economic evaluations are needed to estimate the cost savings of expanding the duration of maternity leave through its impact on breastfeeding and long-term health outcomes. These evaluations could help advocates to strengthen their country’s political will for the extension of maternity leave legislation.

## References

[1] Victora CG, Bahl R, Barros AJ, França GV, Horton S, Krasevec J, et al.; Lancet Breastfeeding Series Group. Breastfeeding in the 21st century: epidemiology, mechanisms, and lifelong effect. Lancet. 2016 1 30;387(10017):475–90. 10.1016/S0140-6736(15)01024-7 26869575

[2] Rollins NC, Bhandari N, Hajeebhoy N, Horton S, Lutter CK, Martines JC, et al.; Lancet Breastfeeding Series Group. Why invest, and what it will take to improve breastfeeding practices? Lancet. 2016 1 30;387(10017):491–504. 10.1016/S0140-6736(15)01044-2 26869576

[3] Global nutrition report 2017: nourishing the SDGs. Bristol: Development Initiatives; 2017. Available from: https://globalnutritionreport.org/documents/2/Report_2017.pdf [cited 2019 Sep 26].

[4] Global nutrition targets 2025: breastfeeding policy brief. Geneva: World Health Organization; 2014. Available from: https://www.who.int/nutrition/publications/globaltargets2025_policybrief_breastfeeding/en/ [cited 2019 Sep 24].

[5] Global breastfeeding scorecard, 2018. Enabling women to breastfeed through better policies and programmes. Geneva: World Health Organization; 2018. Available from: https://www.who.int/nutrition/publications/infantfeeding/global-bf-scorecard-2018.pdf?ua=1 [cited 2019 Sep 23].

[6] Galtry J. The impact on breastfeeding of labour market policy and practice in Ireland, Sweden, and the USA. Soc Sci Med. 2003 7;57(1):167–77. 10.1016/S0277-9536(02)00372-6 12753825

[7] Sinha B, Chowdhury R, Sankar MJ, Martines J, Taneja S, Mazumder S, et al. Interventions to improve breastfeeding outcomes: a systematic review and meta-analysis. Acta Paediatr. 2015 12;104(467):114–34. 10.1111/apa.13127 26183031

[8] Chai Y, Nandi A, Heymann J. Does extending the duration of legislated paid maternity leave improve breastfeeding practices? Evidence from 38 low-income and middle-income countries. BMJ Glob Health. 2018 10 11;3(5):e001032. 10.1136/bmjgh-2018-001032 30364395 PMC6195155

[9] Monteiro FR, Buccini GDS, Venâncio SI, da Costa THM. Influence of maternity leave on exclusive breastfeeding. J Pediatr (Rio J). 2017 Sep-Oct;93(5):475–81. 10.1016/j.jped.2016.11.016 28734689

[10] Ogbuanu C, Glover S, Probst J, Liu J, Hussey J. The effect of maternity leave length and time of return to work on breastfeeding. Pediatrics. 2011 6;127(6):e1414–27. 10.1542/peds.2010-0459 21624878 PMC3387873

[11] Mirkovic KR, Perrine CG, Scanlon KS. Paid maternity leave and breastfeeding outcomes. Birth. 2016 9;43(3):233–9. 10.1111/birt.12230 26991788

[12] Jia N, Dong X-y, Song Y. Paid maternity leave and breastfeeding in urban china. Fem Econ. 2018;24(2):31–53. 10.1080/13545701.2017.1380309

[13] Hamad R, Modrek S, White JS. Paid family leave effects on breastfeeding: a quasi-experimental study of US policies. Am J Public Health. 2018 10 25:e1–3. 30359107 10.2105/AJPH.2018.304693 PMC6301394

[14] Baker M, Milligan K. Maternal employment, breastfeeding, and health: evidence from maternity leave mandates. J Health Econ. 2008 7;27(4):871–87. 10.1016/j.jhealeco.2008.02.006 18387682

[15] Aitken Z, Garrett CC, Hewitt B, Keogh L, Hocking JS, Kavanagh AM. The maternal health outcomes of paid maternity leave: a systematic review. Soc Sci Med. 2015 4;130:32–41. 10.1016/j.socscimed.2015.02.001 25680101

[16] Staehelin K, Bertea PC, Stutz EZ. Length of maternity leave and health of mother and child – a review. Int J Public Health. 2007;52(4):202–9. 10.1007/s00038-007-5122-1 18030952

[17] Maternity and paternity at work. Law and practice across the world. Geneva: International Labour Organization; 2014. Available from: https://www.ilo.org/global/publications/books/WCMS_242615/lang--en/index.htm [cited 2019 Sep 29].

[18] Carroll GJ, Buccini GS, Pérez-Escamilla R. Perspective: what will it cost to scale-up breastfeeding programs? A comparison of current global costing methodologies. Adv Nutr. 2018 9 1;9(5):572–80. 10.1093/advances/nmy041 30060074 PMC6140429

[19] Smith J, Ingham L. Mothers’ milk measures of economic output. Fem Econ. 2005;11(1):41–62. 10.1080/1354570042000332605

[20] Bartick M, Reinhold A. The burden of suboptimal breastfeeding in the United States: a pediatric cost analysis. Pediatrics. 2010 5;125(5):e1048–56. 10.1542/peds.2009-1616 20368314

[21] Bhutta ZA, Das JK, Rizvi A, Gaffey MF, Walker N, Horton S, et al.; Lancet Nutrition Interventions Review Group, the Maternal and Child Nutrition Study Group. Evidence-based interventions for improvement of maternal and child nutrition: what can be done and at what cost? Lancet. 2013 8 3;382(9890):452–77. 10.1016/S0140-6736(13)60996-4 23746776

[22] Holla-Bhar R, Iellamo A, Gupta A, Smith JP, Dadhich JP. Investing in breastfeeding – the world breastfeeding costing initiative. Int Breastfeed J. 2015 2 23;10(1):8. 10.1186/s13006-015-0032-y 25873985 PMC4396713

[23] Shekar M, Kakietek J, Dayton J, Walters D. An investment framework for nutrition. Washington: World Bank; 2017.

[24] Aedo C. [Economic evaluation of prolonging the postnatal period.] Rev Chil Pediatr. 2007;78:10–50. Spanish.

[25] Siregar AYM, Pitriyan P, Walters D, Brown M, Phan LTH, Mathisen R. The financing need for expanded maternity protection in Indonesia. Int Breastfeed J. 2019 6 25;14(1):27. 10.1186/s13006-019-0221-1 31289458 PMC6593591

[26] Dahl GB, Løken KV, Mogstad M, Salvanes KV. What is the case for paid maternity leave? Rev Econ Stat. 2016;98(4):655–70. 10.1162/REST_a_00602

[27] World Bank open data [internet]. Washington: World Bank; 2019. Available from: https://data.worldbank.org/indicator/NY.GDP.PCAP.CD [cited 2019 Jun 17].

[28] ILOSTAT database [internet]. Geneva: International Labour Organization; c2019. Available from: https://ilostat.ilo.org/data/ [cited 2020 March 17].

[29] Addati L, Cassirer N, Gilchrist K. Maternity and paternity at work: law and practice across the world. Geneva: International Labour Organization; 2014.

[30] Global breastfeeding scorecard [internet]. Geneva: United Nationals Children’s Fund; 2017. Available from: https://www.unicef.org/nutrition/index_100585.html [cited 2020 Mar 17].

[31] Stumbitz B, Lewis S, Rouse J. Maternity management in SMEs: a transdisciplinary review and research agenda. Int J Manag Rev. 2018;20(2):500–22. 10.1111/ijmr.12143

[32] Sixty-fifth World Health Assembly. Geneva, 21–26 May 2012 [internet]. Geneva: World Health Organization; 2012. Available from: https://www.who.int/mediacentre/events/2012/wha65/en/ [cited 2019 Oct 1].

[33] [National Household Sample Survey 2015 (PNAD)] [internet]. Río de Janeiro: Brazilian Institute of Geography and Statistics (IBGE); 2015. Portuguese. https://www.ibge.gov.br/en/statistcs/social/population/20620-summary-of-indicators-pnad2.html [cited 2019 Aug 17].

[34] Ghana Living Standard Survey (GLSS7) [internet]. Accra: Ghana Statistical Service (GSS); 2017. Available from: http://www2.statsghana.gov.gh/nada/index.php/catalog/97 [cited 2019 Jun 21].

[35] [National Survey of Demographic Dynamics 2014 (ENADID) [internet]. Aguascalientes: National Institute of Statistics and Geography (INEGI); 2014. Spanish. Available from: https://www.inegi.org.mx/programas/enadid/2014/ [cited 2017 Oct 12].

[36] [National Survey of Occupation and Employment (ENOE)]. Q3-Q4(2013) Q1-Q2(2014) [internet]. Aguascalientes: National Institute of Statistics and Geography (INEGI); 2013. Spanish. Available from: http://en.www.inegi.org.mx/proyectos/enchogares/regulares/enoe/ [cited 2017 Oct 10].

[37] Population estimates and projections 2010–2015 [internet]. Washington: World Bank; 2019 Available from: https://datacatalog.worldbank.org/dataset/population-estimates-and-projections [cited 2019 May 07].

[38] [Intercensal Survey 2015] [internet]. Aguascalientes: National Institute of Statistics and Geography (INEGI); 2019. Spanish. Available from: https://www.inegi.org.mx/programas/intercensal/2015/ [cited 2018 Jan 20].

[39] Ghana Labour Force Survey. Accra: Ghana Statistical Service (GSS); 2015. Available from: http://www2.statsghana.gov.gh/docfiles/publications/Labour_Force/LFS%20REPORT_fianl_21-3-17.pdf [cited 2019 Jun 20].

[40] STPS Article 170: Federal Labor Law [internet]. Mexico City: Department of Labor and Social Welfare; 2019. Spanish. Available from: http://www.stps.gob.mx/bp/secciones/junta_federal/secciones/consultas/ley_federal.html [cited 2019 Sep 29].

[41] Maternity cash benefits for workers in the informal economy [internet]. Geneva: International Labour Organization; 2016. Available from: https://www.ilo.org/beijing/what-we-do/publications/WCMS_537934/lang--en/index.htm [cited 2019 Sep 28].

[42] [Workers’ compensations]. In: Memoria estadística. Chapter 10. Mexico City: Mexican Institute of Social Security (IMSS); 2016. Spanish. Available from: http://www.imss.gob.mx/conoce-al-imss/memoria-estadistica-2014 [cited 2018 Nov 5].

[43] [Brazil Demographic Census 2010 [internet].] Rio de Janeiro: Brazilian Institute of Geography and Statistics (IBGE); 2012. Portuguese. Available from: http://ghdx.healthdata.org/record/brazil-demographic-census-2010 [cited 2019 Aug 17].

[44] Ghana Census 2010. Accra: Ghana Statistical Service (GSS); 2010. Available from: https://www.statsghana.gov.gh/gssmain/storage/img/marqueeupdater/Census2010_Summary_report_of_final_results.pdf [cited 2019 Jun 20].

[45] [Report to the Federal Executive and the Congress of the Union on the financial situation and risks of the Mexican Social Security Institute 2016–2017]. Mexico City: Mexican Institute of Social Security (IMSS); 2017. Spanish.

[46] Mexico. Becoming breastfeeding friendly: a guideto global scale up [internet]. New Haven: Yale School of Public Health; 2018. Available from: https://publichealth.yale.edu/bfci/countries/mexico/ [cited 2010 Mar 30].

[47] Vilar-Compte M, Teruel G, Flores D, Carroll GJ, Buccini GS, Pérez-Escamilla R. Costing a maternity leave cash transfer to support breastfeeding among informally employed Mexican women. Food Nutr Bull. 2019 6;40(2):171–81. 10.1177/0379572119836582 31035773

